# Water-Insoluble Photosensitizer Nanocolloids Stabilized by Supramolecular Interfacial Assembly towards Photodynamic Therapy

**DOI:** 10.1038/srep42978

**Published:** 2017-02-23

**Authors:** Yamei Liu, Kai Ma, Tifeng Jiao, Ruirui Xing, Guizhi Shen, Xuehai Yan

**Affiliations:** 1 State Key Laboratory of Metastable Materials Science and Technology, Yanshan University, Qinhuangdao 066004, P. R. China; 2 Hebei Key Laboratory of Applied Chemistry, School of Environmental and Chemical Engineering, Yanshan University, Qinhuangdao 066004, P. R. China; 3 State Key Laboratory of Biochemical Engineering, Institute of Process Engineering, Chinese Academy of Sciences, Beijing 100190, P. R. China

## Abstract

Nanoengineering of hydrophobic photosensitizers (PSs) is a promising approach for improved tumor delivery and enhanced photodynamic therapy (PDT) efficiency. A variety of delivery carriers have been developed for tumor delivery of PSs through the enhanced permeation and retention (EPR) effect. However, a high-performance PS delivery system with minimum use of carrier materials with excellent biocompatibility is highly appreciated. In this work, we utilized the spatiotemporal interfacial adhesion and assembly of supramolecular coordination to achieve the nanoengineering of water-insoluble photosensitizer Chlorin e6 (Ce6). The hydrophobic Ce6 nanoparticles are well stabilized in a aqueous medium by the interfacially-assembled film due to the coordination polymerization of tannic acid (TA) and ferric iron (Fe(III)). The resulting Ce6@TA-Fe(III) complex nanoparticles (referenced as Ce6@TA-Fe(III) NPs) significantly improves the drug loading content (~65%) and have an average size of 60 nm. The Ce6@TA-Fe(III) NPs are almost non-emissive as the aggregated states, but they can light up after intracellular internalization, which thus realizes low dark toxicity and excellent phototoxicity under laser irradiation. The Ce6@TA-Fe(III) NPs prolong blood circulation, promote tumor-selective accumulation of PSs, and enhanced antitumor efficacy in comparison to the free-carrier Ce6 *in vivo* evaluation.

Photodynamic therapy (PDT) has been regarded as a promising modality for the treatment of a broad range of cancers^1^,^2^. This process involves systemic or local administration of photosensitive drugs, photosensitizers (PSs), followed by localized illumination at the tumor site to activate the PSs using appropriate visible or near-infrared (NIR) light. After being excited, the PS molecules can transfer the excited-state energy to molecular oxygen, thus generating cytotoxic reactive oxygen species (ROS). These locally generated ROS are responsible for destruction of the cellular compartments, leading to tumor cell apoptosis or necrosis^3^,^4^. On the basis of such a destruction process, requiring the combination of three essential components (PS, light, and oxygen), PDT has relatively minimal toxic effects on the biological systems and no repeatability of cumulative toxicity. Therefore, PDT has been an attractive treatment modality against cancer, which improves survival rate without compromising the life quality of oncological patients^5^.

Despite owning many merits, PDT has not yet gained wide clinical acceptance due to certain limitations associated with PSs^6^,^7^,^8^. Most of the PSs, especially those of porphyrin-based PSs (such as Chlorin e6 and Vertiporfin) are highly hydrophobic, resulting in poor water solubility, rapid degradation and clearance in blood circulation, non-selective accumulation, and thus low bioavailability. However, these water-insoluble PSs exhibit a higher accumulation ratio of tumor to normal tissue, compared to hydrophilic PSs, and the hydrophobic characteristic is an important factor affecting the preferential accumulation in cellular hydrophobic domains since these molecules are able to efficiently enter cells by crossing lipid membranes^5^,^9^. Thus, it remains a challenge to rationally design and engineer water-insoluble PSs for enhanced anticancer PDT. Recently, nanoengineering of such kinds of hydrophobic PSs by using nanoscale drug delivery systems (DDS) (nanocarriers) presents an important step forward in overcoming the predicament associated with hydrophobic PSs^10^,^11^,^12^. Nanocarriers are able to exploit the abnormal tumor vasculature via the enhanced permeation and retention (EPR) effect^13^, thus reducing the systemic damage (side effect) and lowering dose of PSs and light. A variety of DDS including liposomes^14^,^15^, micelles^16^,^17^,^18^, polymer nanoparticles^10^, polymer-drug conjugates^19^ and inorganic nanoparticles^20^,^21^ have been developed for the delivery of hydrophobic PSs, offering a tremendous potential as the third generation PS^22^. Although promising, the delivery nanotechnology of PSs is still in its infancy. Development of a high-performance PS delivery system with minimum use of inert materials (non-active components)^23^, or using natural biomolecular material^24^,^25^,^26^,^27^,^28^,^29^ as a carrier for safe and efficient PDT in a simple and green way is highly desired.

Herein, we propose a new strategy for the delivery of hydrophobic PSs with high loading efficiency, associated with the coordination-triggered ultrafast interfacial assembly around nanocores of hydrophobic PSs. In our strategy, Chlorin e6 (Ce6) is selected as a model of hydrophobic PSs. The preparation completed efficiently and instantaneously in mild conditions, without using specialized equipment. The detailed process is illustrated in Fig. 1, Ce6 is firstly aggregated to form the Ce6 nuclei (nanocores) by a precipitate method in an aqueous solution. To reduce the aggregation and further growth into larger crystals of nanocores in the aqueous ambient, coordination-triggered ultrafast coating regarding the interfacial adhesion and assembly, by adding tannic acid (TA) and FeCl_3_ solution, was used to stabilize the formed Ce6 nanocores^30^. TA and FeCl_3_ spontaneously form coordination supramolecular networks around the Ce6 nanocores. The outer complex layer serves as a protection shell, providing the good colloidal dispersion and stability in an aqueous solution. The cellular uptake and laser-triggered phototoxicity of the Ce6@TA-Fe(III) complex nanocolloids were also evaluated in a cell culture medium. The Ce6@TA-Fe(III) complex nanocolloids, which have a high fluorescence emission of Ce6 at a long wavelength of 660–670 nm, in combination with selective accumulation of nanoparticles in tumor tissue, could serve as a clear window and an ideal penetration for imaging-guided PDT *in vivo*.

## Results and Discussion

**Figure 2:**
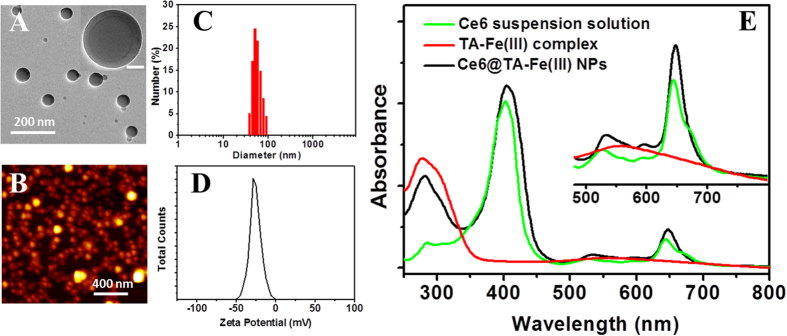
(**A**) TEM image (inset: magnified TEM image, scale bar is 20 nm), (**B**) AFM image, (**C**) Size distribution, and (**D**) Zeta potential of the Ce6@TA-Fe(III) NPs. (**E**) UV/Vis absorption spectra of the complex NPs, Ce6 suspension solution (pH = 6.0) and TA-Fe(III) complex.

Chlorin e6 (Ce6), was one of the most promising PS in the second generation PS with many advantages, such as high photosensitizing efficacy, low dark toxicity and long absorption wavelength^31^. Due to the porphyrin ring structure, the solubilty of Ce6 molecules in the physical condition is very low. However, each Ce6 molecule has three carboxyl groups, the deprotonation/protonation of these carboxyl groups affect the water solubility of Ce6 dramatically^32^. Based on this property, a high yield and environmentally green process was developed to obtain well-dispersion Ce6 nanodrug. Briefly, Ce6 was dissolved in alkaline solution to obtain the ionic forms of Ce6 solution, and then the pH of the above solution was adjusted with the HCl solution (0.1 M) gradually. As the pH value of solution decreased to 8.0, the ionic Ce6 transformed into the aggregated form. The size of the Ce6 nanocores increased during the acidic peptization process (Fig. S1). When the pH was lowered to 6.0, the solution turned into green opalescent suspension. Immediately, TA and FeCl_3_ solutions were added under vigorous stirring, which results in the formation of Ce6@TA-Fe (III) complex nanoparticles (Ce6@TA-Fe(III) NPs). The result indicated that the TA-Fe (III) layer could prevent the initially formed Ce6 nanocores to aggregate into large particles (Fig. S2). The morphology of the as-preparaed Ce6@TA-Fe(III) NPs was determined by transmission electron microscopy (TEM) and atomic force microscope (AFM). As shown in Fig. 2, the Ce6@TA-Fe(III) NPs have a spherical shape and a size of around 60 nm. The magnified TEM image displays the core-shell structure of Ce6@TA-Fe(III) NPs, which is consistent with our previous report^30^. Dynamic light scattering (DLS) measurements revealed that the obtained Ce6@TA-Fe(III) NPs had a relatively narrow size distribution in an aqueous medium (polydispersity index of 0.10), and the hydrodynamic diameter of the Ce6@TA-Fe(III) NPs in water is 60 ± 17 nm, almost consistent with the size determined by TEM and AFM. It has been reported that nanoparticles with a size smaller than 100 nm may be preferentially delivered into tumors due to their enhanced permeability and retention (EPR) effect^33^. The further stability test confirms that the Ce6@TA-Fe (III) NPs could be stable in physiological pH buffers (Fig. S3). In addition, The average ζ-potential of the Ce6@TA-Fe(III) NPs in water was found to be around −25 mV, which could be ascribed to the deprotonation of catechol -OH groups of TA. This negative surface charge of Ce6@TA-Fe(III) NPs may be benificial to avoid their rapid clearance by the mononuclear phagocyte system (MPS)^34^.

UV-vis absorption spectroscopy was used to analyze the the structural organization of the Ce6@TA-Fe(III) NPs (Fig. 2E). The pure Ce6 suspension solution exhibited a strong absorption at 402 nm (Soret peak), and weak Q-bands between 500~700 nm. The blank TA-Fe(III) complex polymer showed an absorption at 280 nm and a weak band around 556 nm. The Ce6@TA-Fe(III) NPs revealed Ce6 peaks superimposing with the absorption curve of TA-Fe(III) complex, indicating the successful coating TA-Fe(III) complex onto the Ce6 nanocores, and also suggesting that there is no changes in the Ce6 chromophore in the Ce6@TA-Fe(III) NPs. The loading efficiency (LE) and encapsulation efficiency (EE) of Ce6@TA-Fe(III) NPs was ~65% and ~90%, respectively, as calculated by UV-vis absorbance (Fig. S4). The high LE may be contributed to the aggregated form of pure PSs soild in the Ce6@TA-Fe(III) NPs.

**Figure 3:**
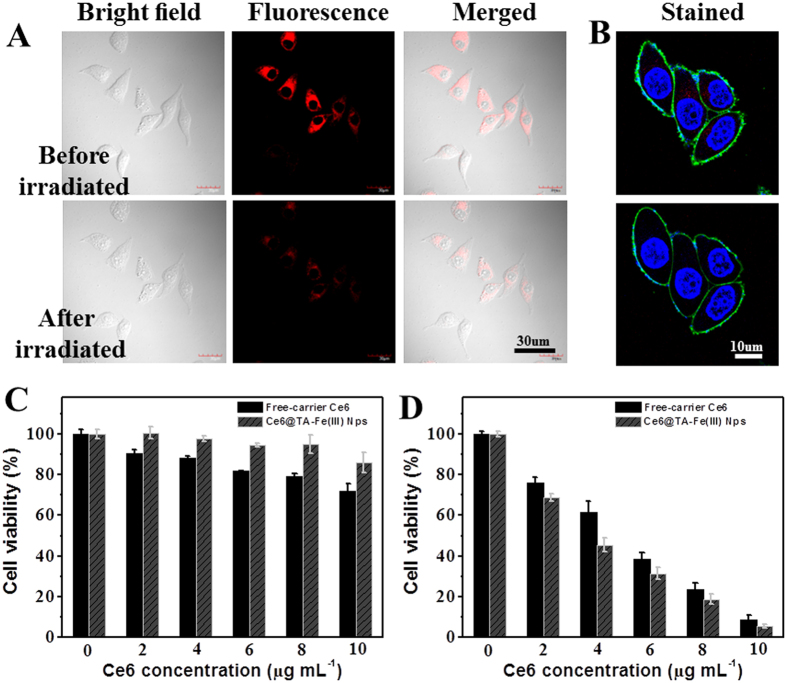
(**A**) Confocal images of MCF-7 cells before and after irradiation with 635 nm laser (Incubation with Ce6@TA-Fe(III) NPs for 12 h). (**B**) Selected frames showing the morphological changes of MCF-7 cells treated with Ce6@TA-Fe(III) NPs under laser irradiation in real time (also see Video S2). The blue nuclei of the living cells stained with Hoechst 33342 (Ex = 405 nm), the green fluorescence is resulted from Alexa Fluor^®^ 488 WGA (Ex = 488 nm) and the red fluorescence from Ce6 (Ex = 635 nm). Cell viability of MCF-7 cells incubation with Ce6@TA-Fe(III) NPs or free-carrier Ce6 at different concentrations for 24 h (**C**) in the dark, (**D**) upon 635 nm laser irradiation (0.1 W cell^−1^, 1 min) and followed by further incubation for 24 h. Data are expressed as means ± S.D. based on three measurements.

The strongly aggregated state of Ce6 in the Ce6@TA-Fe(III) NPs resulted in an inevitable fluorecence quenching due to consumption of excitonic energy by free intramalecular motions (Fig. S5). This aggregated state signigicantly reduced their ROS-generation ability because only monomeric species are photoactive^19^. However, the fluorescence of Ce6 was found to recover gradually after cellular internalization (Fig. S6). As observed by confocal laser scanning microscopy (CLSM), MCF-7 cells presented strong fluorescence signals in the cytoplasm after incubated with the Ce6@TA-Fe(III) NPs for 12 h. This is because the intracellular enviorment contains bio-amphiphilic molecules (such as the lipids of cell membranes). Interaction with these bio-amphiphilic molecules would enhance the solubility of Ce6, and the Ce6 molecules released in the cytoplasm. This intracellular release process may protect Ce6 from leaking and rapid clearance during systemic circulation and ultimately deliver Ce6 into cells, presumably in the cytoplasm. To further identify whether ROS generation ability of the Ce6@TA-Fe(III) NPs was recovered as well, the real-time morphology changes of MCF-7 cells induced by ROS were monitored *in* *situ* by CLSM (Fig. 3A,B, and Video S1–2). MCF-7 cells treated with Ce6@TA-Fe(III) NPs for 12 h revealed typical long spindle morphology and the cell membranes were relatively smooth and intact (Fig. 3A). The bright red fluorescence represented the Ce6 released from the uptaken Ce6@TA-Fe(III) NPs. After irradiation with light, obvious morphology changes were observed. For example, the cells became flat and the membrane turned rough and even collapse. Meanwhile, the fluorescence intensity of Ce6 decreased significantly, which may be photobleached by the produced ROS^35^. Further, the costained experiments were performed to obtain more information in this process. The position of blue and green fluorescence represented the nuclei and the membrane of the cell, respectively. As shown in Fig. 3B, cells turned swollen and round out, which was a typical symptom of cell necrosis and apoptosis^3^,^36^. These results demonstrated that the Ce6 in Ce6@TA-Fe(III) NPs could be released and activated in the cytoplasm, and then damaged the cancer cells upon laser irradiation.

To quantitatively assess the effect of phototoxicity of Ce6@TA-Fe(III) NPs on the cancer cell proliferation *in vitro*, the 3-(4,5-dimethylthiazolyl-2)-2,5-diphenyltetrazolium bromide (MTT) cell survival assay was carried out. In brief, MCF-7 cells were incubated with free-carrier Ce6 and Ce6@TA-Fe(III) NPs for 24 h and then washed with PBS. Next, cells were irradiated with 635 nm laser, and cell viability was determined using the MTT assay (Fig. 3C,D). Both formulations showed negligible cell death within the range of administration dosage without light irradiation, indicating excellent biocompatibility of the Ce6@TA-Fe(III) NPs. After exposure to the λ = 635 nm laser (0.1 W cell^−1^, 1 min), significantly reduced cell viabilities were observed in a concentration-dependent manner. It is worth noting that the Ce6@TA-Fe(III) NPs exhibits lower cell viabilities compared to the free-carrier Ce6 at an equivalent Ce6 dosage. This result suggests that Ce6@TA-Fe(III) NPs generated higher phototoxicity, more efficient than the free-carrier Ce6.

**Figure 4:**
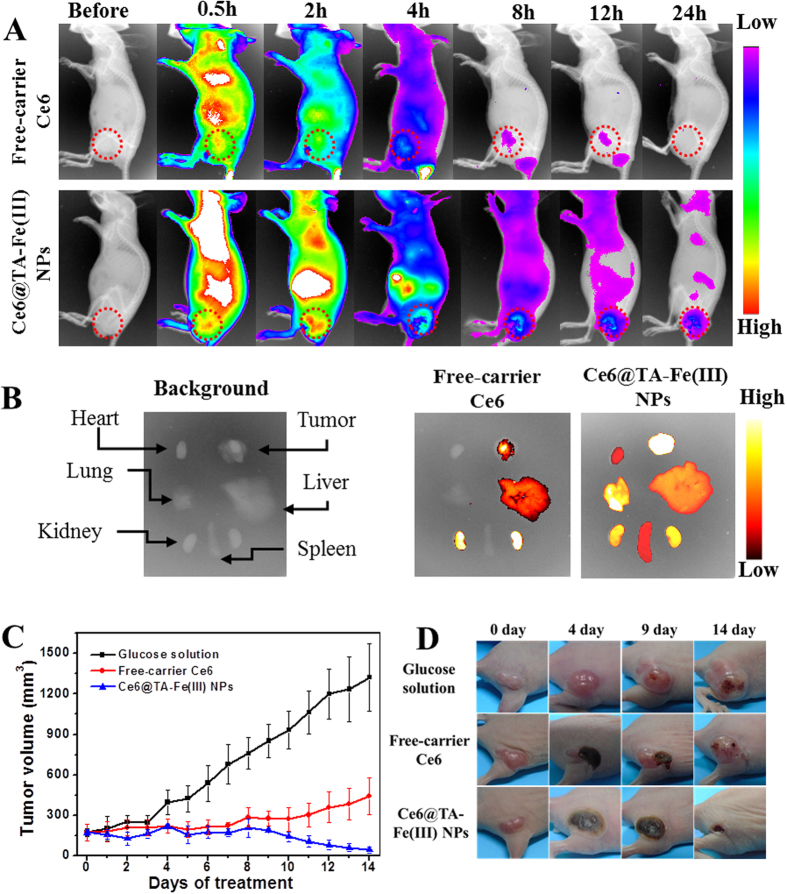
(**A**) Time-dependent whole body fluorescence images of MCF-7 tumor-bearing mice treated with free-carrier Ce6 or Ce6@TA-Fe(III) NPs. Red circles indicate tumor sites. (**B**) *Ex-vivo* fluorescence images of resected organs and tumor from the mice injected with free-carrier Ce6 or Ce6@TA-Fe(III) NPs (24 h post-injection). (**C**) Tumor growth curves of different groups after various treatments. (**D**) Photographs of mice showing the change of tumor after various treatments at different time points.

Precise location and controlled scope of the light irradiation is significantly important to achieve selective therapeutic effect against tumors. That is why the imaging-guided therapy, such as combination of fluorescence imaging and photodynamic therapy, is highly desired. The intrinsic fluorescence of PSs could serve as a bioprobe to imaging the tumor tissue. Based on the intrinsic fluorescence of Ce6, the tumor-imaging ability of Ce6@TA-Fe(III) NPs in human breast tumor-bearing mice was evaluated in a noninvasive manner. After injection of free-carrier Ce6 (4 mg kg^−1^) and Ce6@TA-Fe(III) NPs (4 mg kg^−1^ of Ce6) into the tail vein, the time-dependent biodistribution of Ce6 was monitored using an *in vivo* optical imaging system. As shown in Fig. 4A, when free-carrier Ce6 was injected into the mice, the fluorescence signal was observed primarily in the whole body and then eliminated rapidly, presenting low accumulation in tumor tissue. However, in case of Ce6@TA-Fe(III) NPs-treated mice, after 2 h post-injection, subcutaneous tumor was detected against the surrounding background tissue. In addition, the total fluorescence signal resulting from Ce6@TA-Fe(III) NPs was much higher compared with that of free-carrier Ce6, especially in the tumor tissue. These results suggest prolonged blood circulation and enhanced permeation and retention (EPR) effect of Ce6@TA-Fe(III) NPs. To gain further insight into the biodistribution of Ce6, *ex-vivo* evaluation of excised tissues at 24 h post-injection was performed, Ce6@TA-Fe(III) NPs-treated mice showed a strong fluorescence intensity in the tumor tissue, while that of the control mice was mainly in the kidney (Fig. 4B). This was also confirmed by the semiquantitative data upon *ex-vivo* fluorescence images (Fig. S7). Ce6@TA-Fe(III) NPs exhibited a 2.8-fold higher accumulation in the tumor and 0.6-fold lower accumulation in the kidney, compared to free-carrier Ce6. Moreover, Higher fluorescence intensity of tumor-to-organs were demonstrated in all the excised organs, indicating enhanced tumor selectivity of Ce6@TA-Fe(III) NPs.

According to the results of the enhanced accumulation in tumor tissue, we further investigated the PDT therapeutic efficacy of Ce6 NPs *in vivo*. Nude mice bearing the MCF-7 tumor model were intravenously injected with 5% glucose (as control group), free-carrier Ce6 and Ce6@TA-Fe(III) NPs (equivalent Ce6 2.0 mg kg^−1^ body). After 2 h post-injection, the tumor was irradiated with 635 nm laser (160 mW cm^−2^) for 15 min. As shown in Fig. 4C, mice in the control group experienced a rapid growth of tumor volume, in contrast, the PDT treated groups showed remarkable delays in tumor growth after laser irradiation. However, it is noteworthy that the group treated with Ce6@TA-Fe(III) NPs exhibited better therapeutic effect, compared with free-carrier Ce6 group on day 14. The photographs of tumors during PDT treatment were depicted in Fig. 4D. The tumors of mice in Ce6@TA-Fe(III) NPs group were almost completely inhibited with only scar tissue remained, while the tumors on the free-carrier Ce6 treated group recurred after about one week. These results demonstrated that the Ce6@TA-Fe(III) NPs revealed higher PDT efficacy, attributable to enhanced tumor accumulation of the Ce6@TA-Fe(III) NPs. Besides, we also monitored the body weight changes of the mice for all groups during the treatments, and no obvious body weight loss was observed (Fig. S8), implying that the side effects of the Ce6@TA-Fe(III) NPs were negligible. The above results revealed that the as-prepared Ce6@TA-Fe(III) NPs were very potential in PDT.

In summary, we have developed a new type of nanodrugs of hydrophobic photosensitizers via a simple but robust interfacial coordination assembly, associated with compromise and balance of nucleation of hydrophobic photosensitizers and ultrafast interfacial coating. The obtained Ce6@TA-Fe(III) NPs exhibit a diameter of about 60 nm, negative surface charge and a high loading content of over 65%. The encapsulated Ce6 could selectively release from the complex nanoparticles in the cytoplasm after internalization, subsequently recover its fluorescence and ^1^O_2_ generation capability. The Ce6@TA-Fe(III) NPs has also been demonstrated a selective accumulation in tumor tissue and prolonged blood circulation time, thus leading to an enhanced antitumor PDT. These results suggest that supramolecular interfacial assembly may provide a facile but effective and robust avenue for nanoengineering of hydrophobic photosensitizers towards enhanced PDT.

## Methods

### Materials

Chlorin e6 (Ce6), Tannic acid (TA) were purchased from Sigma-Aldrich, Iron (III) chloride anhydrous (FeCl_3_) and 3-(4,5-dimethylthiazol-2-yl)-2,5-diphenyltetrazolium bromide (MTT) were obtained from Alfa Aesar. Hoechst 33342, and Alexa Fluor^®^ 488 WGA were obtained from Molecular Probes Inc. Unless mentioned otherwise, the cell culture products were supplied by M&C Gene Technology Ltd (Beijing, China). All the chemical reagents were used as received without further purification. High-purity Millipore water (18.2 MΩ) was used throughout the experiments.

### Preparation of Ce6@TA-Fe(III) complex nanoparticles (Ce6@TA-Fe(III) NPs)

Ce6@TA-Fe(III) complex nanoparticles (Ce6@TA-Fe(III) NPs) were prepared as follow: Ce6 was first added to the NaOH solution (pH = 12) to form the ionic form of Ce6 at a concentration of 1 mg mL^−1^. Then, 0.1 M HCl solution was dropped into the ionic Ce6 solution under vigorous stirring. With the decrease of the pH value, the ionic Ce6 transformed into the aggregated form. The nanosized Ce6 cores were stabilized by addition of TA (10 uL, 50 mg mL^−1^) and FeCl_3_ solution(10 uL, 5 mg mL^−1^), respectively. The Ce6@TA-Fe(III) NPs were finally collected and washed by centrifugation.

### Characterization of Ce6@TA-Fe(III) NPs

Dynamic light scattering (DLS) and ζ-potential determinations were performed on a ZetaSizer Nano ZS (Malvern Instruments). The morphology and size of the simples were investigated by transmission electron microscopy (TEM) on a JEM-1011 microscope (JEOL, Japan) and atomic force microscope (AFM) images were collected by FASTSCANBIO (Bruker) in a tapping mode. UV-vis absorption spectra were measured in UV-vis spectrometer (UV-2900, Shimadzu, Japan) equipped with a 1-mm quartz cell. Confocal images were collected on confocal laser scanning microscope (CLSM, Leica TCS SP) using a 60× oil-immersion objective.

### *In vitro* cellular uptake and imaging of Ce6@TA-Fe(III) NPs

MCF-7 cells were routinely cultured in RPMI-1640 medium with 10% fetal bovine serum (FBS). The cells were seeded on 35 mm glass-bottom Petri dishes and allowed to grow overnight before the experiments. The Ce6@TA-Fe(III) NPs were added to the cells and further incubated for 2 h or 12 h. Subsequently, the cells were washed thoroughly with sterile PBS, and then the cells were observed under a CLSM equipped with a 60× oil immersion objective. Images were recorded in fluorescence channel with laser excitation at 635 nm for Ce6. The cytoskeleton and nucleus were stained with Alexa Fluor^®^ 488 WGA and Hoechst 33342 according to the standard protocol provided by the suppliers.

### Cytotoxicity studies of Ce6@TA-Fe(III) NPs

MTT assays were used to assess the cell viability of MCF-7 cells after incubation with the Ce6@TA-Fe(III) NPs upon 635 nm laser irradiation (0.1 W cell^−1^, 1 min). The cells in 96-well plates were incubated with the Ce6@TA-Fe(III) NPs for 24 h in the dark. After incubation, the cells were washed with PBS and exposed to laser irradiation. The cells were further incubated in fresh medium for 24 h and washed with PBS. Then MTT in PBS solution (100 *μ*L, 0.5 mg mL^−1^) was added into each well. After incubation for 4 h, the supernatant was discarded and the precipitate was dissolved in DMSO (100 *μ*L) with gentle shaking. The absorbance of MTT at 570 nm was monitored by the microplate reader. Free-carrier Ce6 was dissolved in DMSO for the *in vitro* PDT experiments for comparative study. Immediately before use, it was diluted with culture medium to the desired concentrations. The final concentration of DMSO did not exceed 1% (v/v). The cells without any treatment were used as control. All experiments were performed in triplicate, and all the data were presented as the averaged results and standard deviation.

### *In vivo* NIRF imaging of Ce6@TA-Fe(III) NPs

Female BALB/c nude mice (4–6 w, 16–18 g) were purchased from Vital Laboratory Animal Center (Beijing, China). The human xenograft model was prepared by subcutaneously injecting a suspension of MCF-7 cells (100 *μ*L, 5 × 10^7^ cells mL^−1^) in sterilized PBS at the right hind leg. Approximately one week after inoculation, the tumors were well-established. Mice were intravenously injected with freshly prepared Ce6@TA-Fe(III) NPs or free-carrier Ce6. Fluorescence signals were recorded before and 0.5, 2, 4, 8, 12, and 24 h after injection using the Kodak animal imaging system (Kodak, USA. Excitation filter: 650 nm, Emission filter: 700 nm). To evaluate the imaging results, a region of interest (ROI) was drawn around the brain region. Student’s t-test was used to calculate P values.

### Photodynamic Therapeutic efficacy of Ce6@TA-Fe(III) NPs in MCF-7 tumor-bearing Mice

To evaluate PDT efficacy *in vivo*, human xenograft tumor models were established as described above. When the average tumor volume reached about 170 mm^3^, nude mice were randomly grouped into three groups (n = 5). 5% glucose, free-carrier Ce6 or Ce6@TA-Fe(III) NPs with an equivalent Ce6 dose of 2 mg kg^−1^ were administered through tail vein intravenously injection. At 2 h post-injection, the tumor site of mice was irradiated with 635 nm laser (160 mW cm^−2^) for 15 min. The PDT efficacy was evaluated by tumor volumes, which was calculated by the following equations: Tumor volume (mm^3^) = 0.5 × length × width^2^. The body weight was as well recorded every day. All the above animal experiments were carried out under the relevant guidelines and approved by the Institutional Ethical Committee of Animal Experimentation of Institute of Process Engineering (Chinese Academy of Sciences).

### Statistical analysis

All data are reported as the means ± the standard deviation (SD) unless otherwise stated. Means were compared using student’s t test. The results were considered statistically significant, if two-tailed P-values were less than 0.05.

## Additional Information

**How to cite this article**: Liu, Y. *et al*. Water-Insoluble Photosensitizer Nanocolloids Stabilized by Supramolecular Interfacial Assembly towards Photodynamic Therapy. *Sci. Rep.* **7**, 42978; doi: 10.1038/srep42978 (2017).

**Publisher's note:** Springer Nature remains neutral with regard to jurisdictional claims in published maps and institutional affiliations.

## Supplementary Material

Supporting Information

Supplementary Movie S1

Supplementary Movie S2-1

Supplementary Movie S2-2

## Figures and Tables

**Figure 1:**
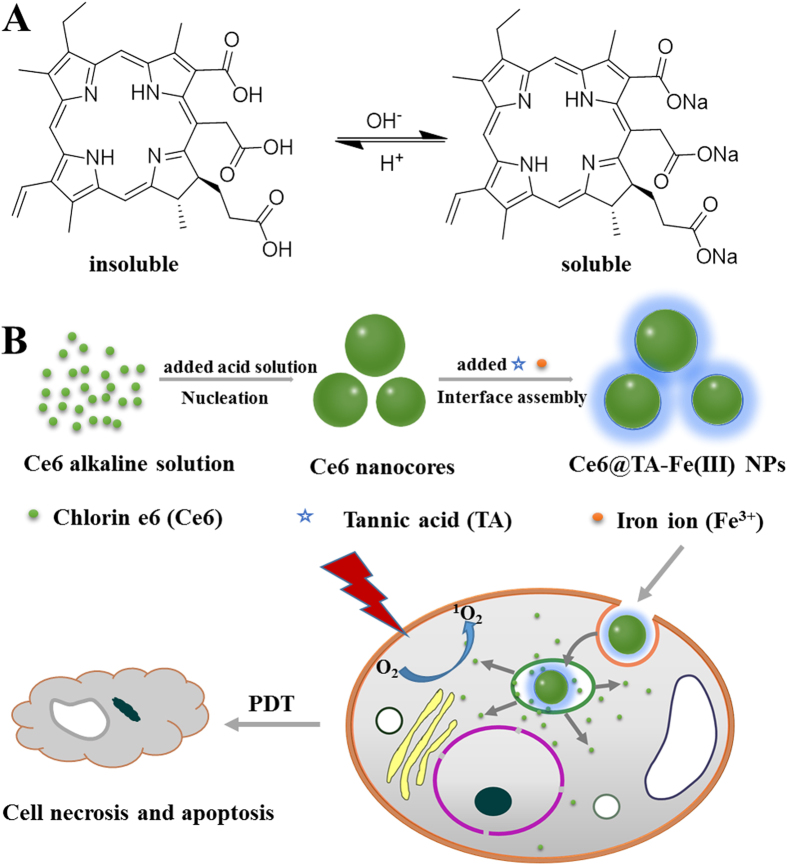
(**A**) The structure forms of Ce6 with pH change. (**B**) Schematic illustration for the fabrication of the Ce6@TA-Fe(III) NPs towards PDT therapy.
