# Supplementary material for: Water-Insoluble Photosensitizer Nanocolloids Stabilized by Supramolecular Interfacial Assembly towards Photodynamic Therapy
Source: Sci Rep. 2017 Feb 23;7:42978. doi: 10.1038/srep42978 (PMC5322353; doi:10.1038/srep42978)
Supplement: Supporting Information [file srep42978-s1.pdf]

Supplementary Information

Water-Insoluble Photosensitizer Nanocolloids Stabilized by Supramolecular Interfacial Assembly towards Photodynamic Therapy

Yamei Liu[1,2], Kai Ma[1,2],Tifeng Jiao[1,2,*], Ruirui Xing[1,2,], Guizhi Shen[3,*], Xuehai Yan[3,*]

[1] State Key Laboratory of Metastable Materials Science and Technology, Yanshan University, Qinhuangdao 066004, P. R. China

[2] Hebei Key Laboratory of Applied Chemistry, School of Environmental and Chemical Engineering, Yanshan University, Qinhuangdao 066004, P. R. China

[3] State Key Laboratory of Biochemical Engineering, Institute of Process Engineering, Chinese Academy of Sciences, Beijing 100190, P. R. China

Corresponding Author

∗Authors to whom correspondence should be addressed.

Email: tfjiao@ysu.edu.cn; gzshen@ipe.ac.cn; yanxh@ipe.ac.cn.

Homepage: www.yan-assembly.org

Supplementary information

To test the colloidal stability of Ce6@TA-Fe(III) NPs in the physiological pH buffers. DLS measurements of Ce6@TA-Fe(III) NPs in 0.1× PBS and DMEM at 25 ^{o}C were monitored over a week period.

To test the Ce6 loading efficiency (LE) and encapsulation efficiency (EE), the Ce6@TA-Fe(III) NPs were lyophilized and weighed, and then dissolved in an alkaline solution. The concentration of Ce6 was calculated according to UV absorbance at 402 nm and referring to a standard curve of free-carrier Ce6 concentration in an alkaline solution (Fig. S4). The LE and EE were calculated by the following equations:

LE (%) =amount of Ce6 in Ce6@TA-Fe(III) NPs / amount of Ce6@TA-Fe(III) NPs × 100%;

EE (%) =amount of Ce6 in Ce6@TA-Fe(III) NPs / amount of Ce6 added × 100%

Fluorescence spectra of free-carrier Ce6 and Ce6@TA-Fe(III) NPs were measured with a spectrofluorometer (F-4600, Hitachi, Japan).

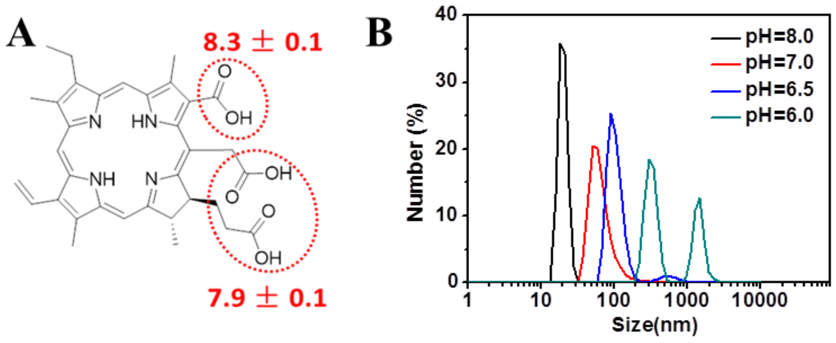

Figure S1. (A) Red number indicated the pK_a values of chlorin carboxylate groups,which were determined by ^{13}C NMR in the Ref S1. (B) Size distribution of free-carrier Ce6 in aqueous solutions at different pH.

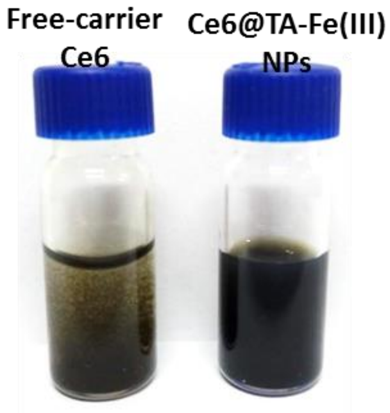

Figure S2. Photograph of the free-carrier Ce6 (left) and Ce6@TA-Fe(III) NPs (right) in the aqueous solution of pH=6.0.

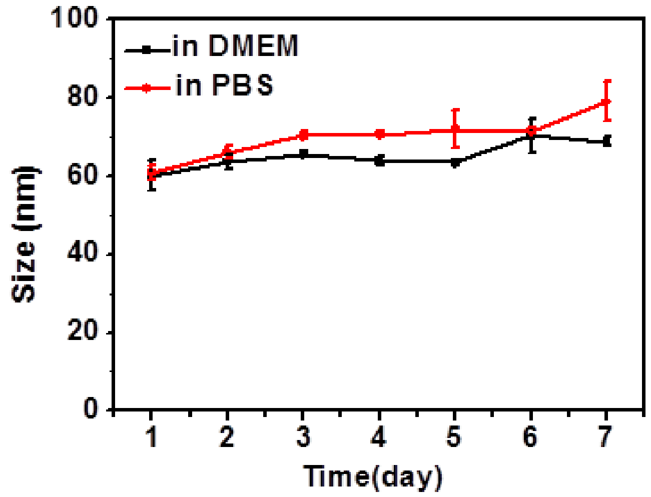

Figure S3. Colloidal stablity of Ce6@TA-Fe(III) NPs in 0.1× PBS and in DMEM.

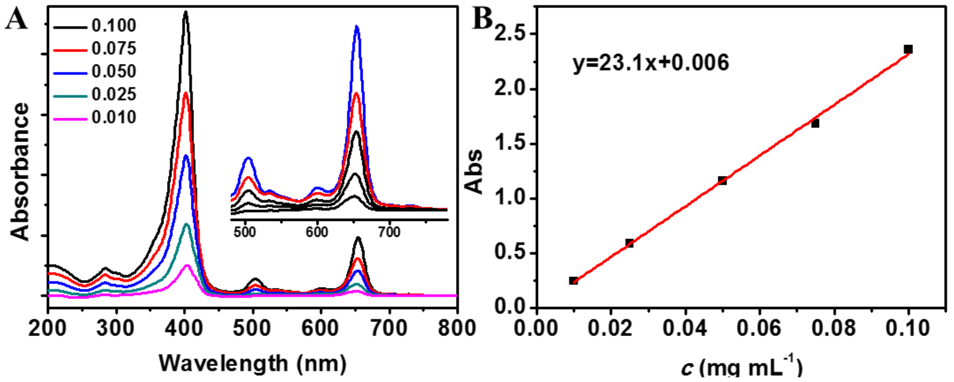

Figure S4 (A) The UV-vis spectrum of pure Ce6 in alkaline solution (pH=11) at different concentration. (B)The standard curve of Ce6 constructed from UV-Vis measurements at peak 402 nm.

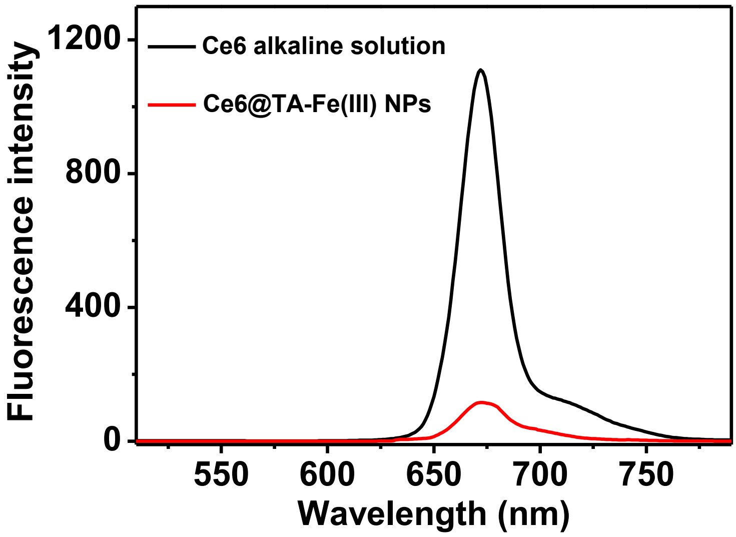

Figure S5. Fluorescent spectra for Ce6 alkaline solution (pH=11.0) and Ce6@TA-Fe(III) NPs with the same concentration of Ce6 (excited at 402 nm).

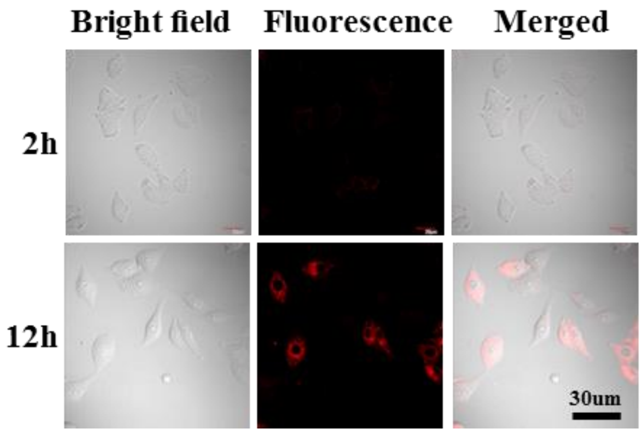

Figure S6. CLSM images of MCF-7 cells incubated with Ce6@TA-Fe(III) NPs at various time.

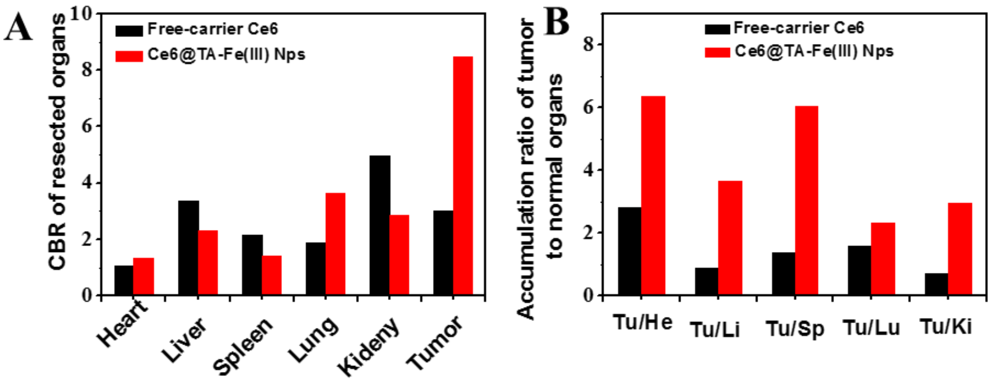

Figure S7. (A) Semi-quantitative bio-distribution of in various organs as determined by the fluorescence intensities and (B) Tumor-to-organs ratio for free-carrier Ce6 solution and Ce6@TA-Fe(III) NPs at 24 h post-injection.

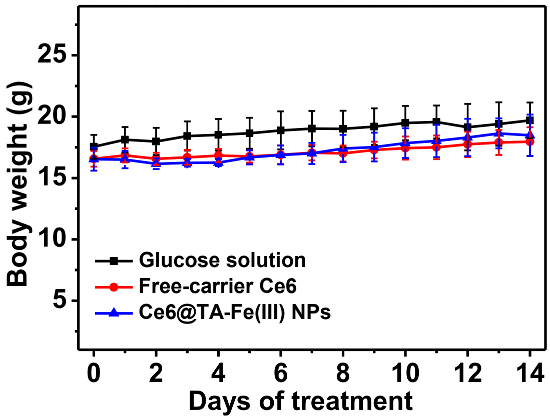

Figure S8. The body weight of MCF-7 xenograft tumor-bearing mice monitored during the PDT treatment. Data are presented as mean±S.D (n=5).

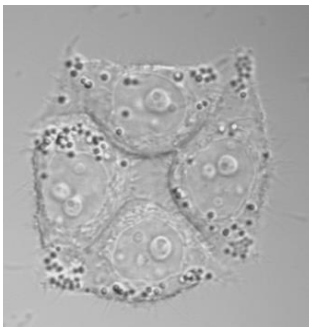

Video S1. Dynamic process of morphological changes of MCF-7 cells (control) under irradiated conditions.

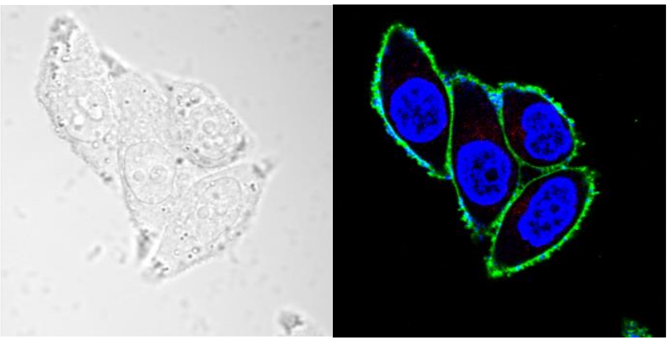

Video S2. Dynamic process of morphological changes of MCF-7 cells cultured with Ce6@TA-Fe(III) NPs under irradiated conditions (left: Bright field; right: Fluorescent channel).

Reference:

1. Vermathen, M., Marzorati, M., Vermathen, P. & Bigler, P. pH-Dependent Distribution of Chlorin e6 Derivatives across Phospholipid Bilayers Probed by NMR Spectroscopy, *Langmuir*, **26**, 11085-11094 (2010).
